# Harnessing Context Sensing to Develop a Mobile Intervention for Depression

**DOI:** 10.2196/jmir.1838

**Published:** 2011-08-12

**Authors:** Michelle Nicole Burns, Mark Begale, Jennifer Duffecy, Darren Gergle, Chris J Karr, Emily Giangrande, David C Mohr

**Affiliations:** ^3^Audacious SoftwareChicago, ILUnited States; ^2^Department of Communication StudiesNorthwestern UniversityEvanston, ILUnited States; ^1^Department of Preventive MedicineFeinberg School of MedicineNorthwestern UniversityChicago, ILUnited States

**Keywords:** Depression, behavior therapy, telemedicine, mobile health, mobile phone, cellular phone, sensors, data mining, artificial intelligence, context-aware systems

## Abstract

**Background:**

Mobile phone sensors can be used to develop context-aware systems that automatically detect when patients require assistance. Mobile phones can also provide ecological momentary interventions that deliver tailored assistance during problematic situations. However, such approaches have not yet been used to treat major depressive disorder.

**Objective:**

The purpose of this study was to investigate the technical feasibility, functional reliability, and patient satisfaction with Mobilyze!, a mobile phone- and Internet-based intervention including ecological momentary intervention and context sensing.

**Methods:**

We developed a mobile phone application and supporting architecture, in which machine learning models (ie, learners) predicted patients’ mood, emotions, cognitive/motivational states, activities, environmental context, and social context based on at least 38 concurrent phone sensor values (eg, global positioning system, ambient light, recent calls). The website included feedback graphs illustrating correlations between patients’ self-reported states, as well as didactics and tools teaching patients behavioral activation concepts. Brief telephone calls and emails with a clinician were used to promote adherence. We enrolled 8 adults with major depressive disorder in a single-arm pilot study to receive Mobilyze! and complete clinical assessments for 8 weeks.

**Results:**

Promising accuracy rates (60% to 91%) were achieved by learners predicting categorical contextual states (eg, location). For states rated on scales (eg, mood), predictive capability was poor. Participants were satisfied with the phone application and improved significantly on self-reported depressive symptoms (beta_week_ = –.82, *P* < .001, per-protocol Cohen *d* = 3.43) and interview measures of depressive symptoms (beta_week_ = –.81, *P* < .001, per-protocol Cohen *d* = 3.55). Participants also became less likely to meet criteria for major depressive disorder diagnosis (b_week_ = –.65, *P* = .03, per-protocol remission rate = 85.71%). Comorbid anxiety symptoms also decreased (beta_week_ = –.71, *P* < .001, per-protocol Cohen *d* = 2.58).

**Conclusions:**

Mobilyze! is a scalable, feasible intervention with preliminary evidence of efficacy. To our knowledge, it is the first ecological momentary intervention for unipolar depression, as well as one of the first attempts to use context sensing to identify mental health-related states. Several lessons learned regarding technical functionality, data mining, and software development process are discussed.

**Trial Registration:**

Clinicaltrials.gov NCT01107041; http://clinicaltrials.gov/ct2/show/NCT01107041 (Archived by WebCite at http://www.webcitation.org/60CVjPH0n)

## Introduction

Major depressive disorder affects nearly 7% of the population annually [1] and is the leading cause of disease burden in middle- and high-income countries worldwide [2]. Individuals with major depressive disorder have higher medical costs [3], exacerbated medical conditions [4], and mortality almost twice that of nondepressed people [5]. Thus, major depressive disorder poses an extraordinary public health problem in terms of prevalence, cost, morbidity, and mortality.

While psychological treatments for depression can be effective [6], they are often plagued by access barriers and high rates of attrition [7,8]. Internet interventions have been touted as an antidote to access barriers, but they appear to produce more modest outcomes [9], in part also due to high attrition [10]. Mobile phones, however, have penetrated nearly all strata of society [11,12] and hold promise as a ubiquitous treatment platform through which the connection between patient and intervention is continuous and reciprocal.

At least two broad classes of treatment components can be delivered via mobile phone. First, these platforms offer the opportunity to deliver interactive tools to patients in their environment. These tools, sometimes referred to as ecological momentary intervention [13], can prompt patients to input information about their situation or internal states, and provide in-the-moment responses personalized to a patient’s immediate needs. While such interventions have been explored with regard to health behaviors and severe mental illness (eg, bipolar disorder [14]), recent reviews reveal no such work in unipolar depression [15,16].

Second, mobile systems also have the potential to apply machine learning techniques that can monitor and learn to recognize a patient’s circumstances and state. Smartphones contain numerous sensors (eg, global positioning system [GPS], Bluetooth) that could provide clues to patient states and contexts. Smartphones also have the ability to conduct ecological momentary assessment and allow patients to report, or “label,” their current states. Machine learning, or data mining techniques, can be used to automatically learn the relationship between these two sources of data. This relationship is captured in what is known as a “learner” that can then be used to develop individualized predictions of patient states solely from low-level sensor data. Once trained using ecological momentary assessment data, the learner could potentially identify patient states continuously and passively, with little effort from the patient. Sensor-based awareness of patient states would enable context-appropriate clinical responses [18,19] (eg, delivering timely feedback, providing guidance during distress or problematic situations, or intervening on nonadherence to homework) without relying on the patient to initiate such therapeutic interactions.

A few such context-aware systems have been developed and tested in mHealth interventions. The Intel Mobile Heart Health prototype uses data from sources such as mobile phone-based ecological momentary assessment and a small electrocardiograph sensor with accelerometer to detect changes in heart rate variability, activity, and mood. If individualized threshold values are reached, the mobile phone delivers cognitive behavioral and mindfulness techniques designed to reduce stress [20]. Although used for social networking rather than health care purposes, the CenceMe mobile phone application has achieved promising accuracy rates within a small sample in predicting socially relevant states such as whether the user was conversing [21]. CenceMe also used such data to generate higher-level descriptions of users’ recent behavioral or lifestyle patterns (eg, “party animal,” which was determined through presence at parties and number of social interactions). Learners detected users’ basic activity level (eg, sitting, running). In the e-SENSE [22] project, body sensors allowed collection of a rich set of physiological data used to infer mood. Predicted mood was then integrated into users’ instant messaging chats. However, the required electrodes and belt did not approach the convenience of a context-sensing platform that relies solely on mobile phone sensors. These projects are in very early stages of development, and we are aware of no trials that formally evaluated clinical outcomes.

The current study aimed to maximize the potential of mobile interventions to target depression. We developed a mobile phone intervention, called Mobilyze!, that includes the capacity to deliver ecological momentary intervention. We also developed and piloted a context-aware system to identify participants’ location, activity, social context, mood, emotions, and cognitive/motivational states. The mobile intervention was supported by a website, which allowed access to lessons, tools, and graphical feedback on participants’ states. The intervention was supported by a manualized telephone coaching protocol [23,24] to enhance adherence. 

The aims of this field trial were to evaluate the technical feasibility and reliability, functional reliability, and acceptability of this system. Secondarily, we measured depression outcomes, as well as changes in anxiety symptoms due to their frequent co-occurrence with depression.

## Methods

### Study Design

This was a single-arm field trial of Mobilyze!, an 8-week multimodal intervention for depression, that included 1) mobile phone sensing and ecological momentary intervention, 2) an interactive website for behavioral skills training, and 3) email and telephone support from a coach assigned to each participant. The trial was approved by the institutional review board at Northwestern University (Chicago, IL, USA).

### Participants

Participants were recruited through online advertising venues such as Google AdWords and Craigslist. The advertisements directed individuals to the lab webpage, where interested individuals completed an online screener and provided their contact information. Those who met initial criteria were scheduled for a telephone eligibility interview and emailed a link to a digital consent form. At the beginning of this interview, staff discussed with participants each section of the consent form, which included sections informing users as to how their mobile phone sensor data would be collected, de-identified, used, and stored. Participants’ verbal consent was then obtained. Receipt of the electronically signed digital consent form was also required prior to enrollment in the trial.

Inclusion criteria were a diagnosis of major depressive disorder using the telephone-administered Mini-International Neuropsychiatric Interview [25,26], a score of 11 or more (Table 3 in [27]) on the 16-item Quick Inventory of Depression Symptoms–Clinician Rated (QIDS-C) [28], and a score of 10 or more [29] on the 8-item Patient Health Questionnaire (PHQ-8) [30]. All participants spoke and read English, were at least 19 years of age, lived in the United States, reported being within a cellular network range most of the day, and reported having an email account, computer, broadband Internet access, and comfort using the Internet and mobile phones. Exclusion criteria were sensory impairments that would prevent participation, dementia (defined as scoring <25 on the Telephone Interview for Cognitive Status [31]), current participation in psychotherapy, initiation of an antidepressant medication in the past 10 days, or a severe psychiatric condition, as measured by the Mini-International Neuropsychiatric Interview, that rendered the intervention inappropriate (eg, psychotic disorders, bipolar disorders, severe agoraphobia, severe suicidality, and current harmful levels of substance or alcohol use).

### Treatment

The treatment model was based on a behavioral activation approach [32,33] involving engagement in positive activities, experimentation with behavioral coping strategies, and use of such coping skills to reduce depressogenic avoidance behaviors. The mobile phone was used to translate this therapy into real time and to pilot a context-aware system that aimed to passively (ie, without requiring patient initiation) identify and respond to patient states.

Participants completed 8 weeks of the Mobilyze! intervention. Enrolled participants received at no charge 1) temporary use of a Nokia 5800 XpressMusic mobile phone with accessories, which cost the study US $547.88 per unit, 2) a cellular service plan, including voicemail and unlimited data usage, voice minutes, and text messaging, through T-Mobile at US $50 per month, per participant, and 3) login credentials to the website. Mobile phones were mailed to participants or made available for in-person pick up, according to participant preference. After the intervention concluded, phones were returned by participants via prepaid envelopes. 

#### Context-Aware System

**Figure 1:**
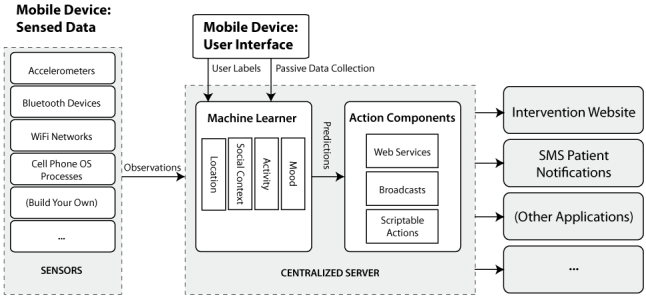
A mobile phone-driven context-aware system (OS = operating system, SMS = short message service)

Our context-aware system used an architecture comprising 3 phases [34] (see Figure 1). In phase 1, sensors housed on the mobile phone gathered observations about the participant and their environment. These observations were transmitted to a secure server hosting a learner. In phase 2, the learner used an algorithm to inductively “learn” the relationship between sensor data and the participant’s reported social context, activity, location, and internal states [35]. The learner could then predict participant states based on sensor data, and these predictions were passed to the action components in phase 3. The action components provided mechanisms for relaying predictions to other external outreach applications. In the current study, the action component consisted of the phone application itself, which displayed predicted states to the participant. The architecture, however, can be extended in the future to trigger outreach events based on the predictions (eg, sending a short message service [SMS, ie, text] notification, updating a feedback graph on a website, or notifying the coach via email).

In this project, we used the mobile phone as the primary sensing platform. We also made use of a service-oriented architecture [36] for context-aware computing [37]. While the mobile phone provided the primary sensing platform, it transmitted sensor data securely via encrypted, password-protected tunnels to a variety of server-based components that provided logging, learning, and prediction services. Since these components communicated using extensible messaging and presence protocol (XMPP) [38], a common network protocol, this design allowed us to distribute the individual components among various hardware servers (providing scalability) while providing a common protocol for adding additional services (providing extensibility). We will now discuss the specific implementation of the context-aware system used in the current study.

##### Phase 1: Data Collection

Contextual data were acquired from a collection of 38 sensors (see Multimedia Appendix 1) or more depending on the number of proximal Bluetooth devices or open applications on the mobile phone. Some of these data were collected directly from phone sensors, including GPS, Wi-Fi, Bluetooth detection of other wireless devices (eg, personal computers, some video game consoles), accelerometer, and ambient light. Other contextual data were inferred by the phone application from information available on the phone. This included time/day and activities of the phone’s operating system (eg, recent calls, active phone applications).

The phone application transmitted encrypted sensor data to the secure backend server, via the XMPP interface protocol. The backend server logged the information by inserting it into a database. The backend also sent the sensor data to the server housing the learners, again using XMPP to lend the backend greater extensibility.

##### Phase 2: Learners

**Figure 2:**
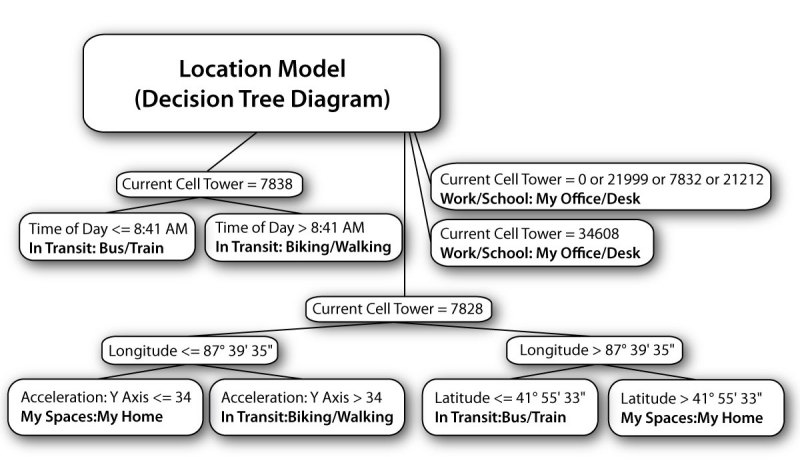
Decision tree model predicting location from sensor values, generated from a research staff member’s state ratings and sensor data (potentially identifying information has been altered)

Participants were periodically prompted to self-report their states using ecological momentary assessment on the phone. Sensor data acquired at these times were paired with simultaneously labeled state data. Using the labeled data, individualized prediction models were generated to identify specific user states from sensor values, including location, activity, social environment, and internal states (eg, mood, concentration). For every state, a machine learning algorithm generated a participant-specific model to predict that state from sensed data in the future. The machine learning algorithms discarded irrelevant information that did not improve the predictive value of the model, using data partitioning and averaging for continuous states, and information theory measures of information gain and entropy [39] for categorical states. States operationalized by scales (eg, sadness) were predicted using regression trees (ie, pruned Weka [17,40] REPTrees, version 3.6; Machine Learning Group, University of Waikato, Hamilton, New Zealand). Categorical states (eg, location) were predicted using J48 classifiers, which are Weka’s adaptation of C4.5 decision trees [41] (see Figure 2). Pruning was also enabled for the location models. For the remaining categorical models, pruning was disabled due to their binary nature (eg, whether the user is having a “task-related conversation” or not). 

##### Phase 3: Action Components

Every 5 minutes, the mobile phone application sent current, unlabeled sensor values to the backend. The learner used these sensor readings to make predictions and infer the participant’s state without input from the participant. Because we rely on a service-oriented architecture, predicted states could be used to implement context-aware functionality through multiple devices. As the accuracy of predictions could not be determined prior to the trial, predictions were only used to display the currently predicted states to users on the mobile phone, as opposed to triggering any outreach or intervention.

When participants viewed the state entry ecological momentary assessment forms on the mobile phone, predictions as to their current states prepopulated the responses. A message at the bottom of the screen, in orange text, notified users of the date and time at which the predicted values were generated. If the phone lacked connectivity at that time, the user’s last self-reported states were used to prepopulate the responses, and a green message indicated the date and time at which the user labeled these previous states.

#### Training the Context-Aware System

Development of accurate predictions requires training. Participants were periodically requested to label their states by selecting values for each context category from a drop-down menu on the mobile phone [42]. For example, if a participant is gardening outside while their family is inside the house, she or he might rate physical activity level as “light,” location as “my yard/porch,” conversation type as “none,” and social context as “alone” in their immediate vicinity but “with family member(s)” in the wider environment. The learner would then match the sensed data to these state labels. Every time participants entered their states (this was described to participants as “training the phone”), new models were generated to accommodate the new data.

**Figure 3:**
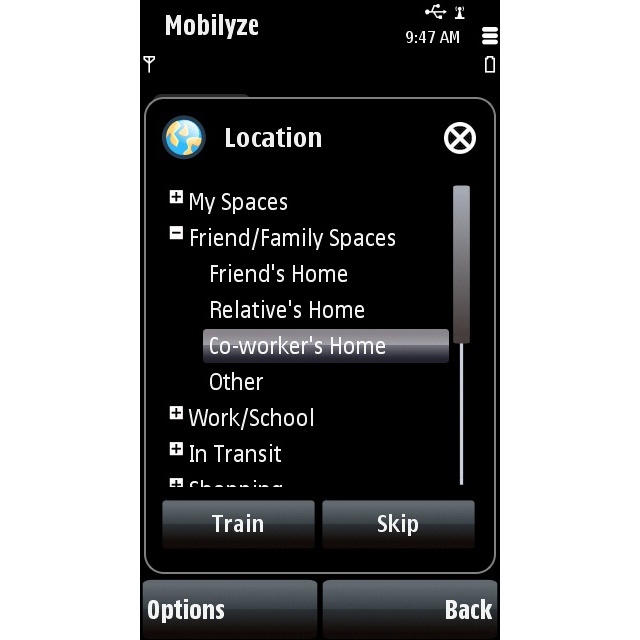
Screenshot, ecological momentary assessment of location on the mobile phone

Specifically, the mobile phone application prompted users to report their current states 5 times per day or more according to participant preference. Participants could also label their states at any time on their own initiative. Using a 7-point Likert scale, participants rated their overall mood, intensity of discrete emotions (ie, happiness, sadness, anger, and anxiety), fatigue, pleasure, sense of accomplishment, concentration and engagement, and perceived control over current activities. Physical exertion was rated on a 4-point scale. Participants selected their location from options including a variety of public and private spaces (eg, their home, a friend’s home, their office/desk at work, or a bus/train; see Figure 3). Participants’ interactions with others were rated according to the type of conversation in which they were engaged, if any (eg, casual, task-related, disagreement, or none), as well as their relationship to others (eg, friends, family, strangers, or pets) in their immediate vicinity and larger environment. Such prompts occurred at random times between 7 AM and 10 PM, and participants could modify these hours via the website.

When participants were prompted to enter their states, the application saved the current values of their phone sensors. Once participants had finished reporting their states, their responses were paired with the sensor data and sent to the backend server for processing by the learner.

#### Ecological Momentary Interventions

Additionally, the mobile phone application used local storage to combine mood ratings entered on the phone with those entered on the website and, consequently, derive a range, average, and standard deviation of mood. Participants received tailored feedback using these values. For example, when a user’s self-reported mood was outside her or his typical range, a message would appear on the phone designed to reinforce improvement or suggest using a tool (see Website, below) in the case of deterioration. Other local data were used to provide feedback in such conditions as a substantial change in the participant’s mood since their previous rating.

To support adherence to therapeutic activities scheduled via tools on the website, participants could choose to receive reminders on their mobile phones prior to the scheduled start times. Each reminder was followed by a check-in prompt after the activity was scheduled to end. If users indicated they had not completed a scheduled task, they received follow-up questions via a troubleshooting feature. Obstacles to completing the task (eg, lack of motivation) were identified per responses to these follow-up questions, and users were then provided with guidance on overcoming their specific difficulties.

#### Website

The website served several functions. It provided a medium with which both participants and coaches could visualize state data entered into the mobile phone application. It also contained an interface allowing coaches to view the activity of their patients, including logins, lessons completed, and states labeled on the phone.

On the website, 9 didactic lessons that taught principles of behavioral activation were available. The lessons could be accessed through a computer or the mobile phone. These lessons were released weekly and required approximately 15 minutes to read. The initial lesson included psychoeducation on depression and detailed instructions on training the mobile phone. Subsequent lessons taught participants how to monitor the effect of daily behaviors on mood, schedule positive activities, experiment with new responses to distressing situations, recognize and change avoidance patterns (eg, rumination and procrastination), and maintain gains after treatment. Each lesson was paired with an interactive tool to provide participants with opportunities to apply the treatment concepts discussed in the lesson. Examples include an activity calendar with which participants could monitor and schedule their activities, as well as tools leading participants through each step involved in designing behavioral experiments or formulating tailored plans to replace avoidance behaviors with active coping behaviors. These tools were designed to be completed in just a few minutes, and participants were instructed to use the current week’s tool on a daily basis.

**Figure 4:**
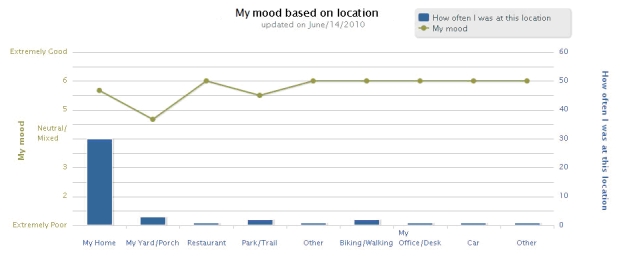
Graphical feedback available to users on the website (blue bars denote locations that a participant reported on the mobile phone, and the frequency with which each location was reported; the green line denotes the participant’s average reported mood in each location)

Interactive graphical feedback tools displayed data from the backend server, which replicated and reformatted participant-labeled data and concurrent sensor values. These were transmitted to the Web server, which hosted the intervention website as well as its own database. Thus, participants could graph their ratings of emotions and other subjective experiences, and explore how these ratings were related to their reported locations (see Figure 4). The aim was to help participants better understand how they spend their time, and identify behaviors they would like to increase or decrease. Participants could also access a graph of their mood over a selected time period (ranging from the last 7 days to the duration of the study) to view their progress. Furthermore, coaches could create graphs associating any 2 states (eg, mood against level of physical exertion, mood against fatigue) to help participants identify patterns.

#### Human Support

A manualized telephone coaching protocol [23] was used to enhance adherence via principles of supportive accountability [24]. Supportive accountability is a model of support that involves collaborative goal setting for adherence, as well as monitoring and encouragement in the context of a supportive relationship. This support model has been previously piloted in an Internet intervention [43]. Coaches also elicited usability feedback on Mobilyze! and provided a point of contact for difficulties. The first coaching call lasted approximately 45 minutes with the aim to establish rapport, address participant concerns or questions, and elicit and reinforce the participant’s motivations for learning to manage depression. Participants then received a 5- to 10-minute coaching call and at least 1 email weekly. Emails were tailored to participants’ mobile phone and website usage patterns, which coaches could monitor online. Participants also received a 30- to 45-minute technical demonstration of the mobile phone (in person or over the telephone) by the lab software engineer or study coordinator. While supportive accountability is designed to be applied by non-mental health providers, the coaches in this trial were PhD-level clinicians given the novelty of the intervention. The author of the coaching manual led weekly group supervision meetings.

### Outcome Measures

Outcomes were assessed at baseline, week 4 (midtreatment), and week 8 (posttreatment). Participants were compensated $20 for each of the first 2 assessments and $50 for the final assessment and return of the mobile phone. Interview measures were administered over the telephone, while self-report measures were administered via a secure online questionnaire hosting service.

The telephone-administered Mini-International Neuropsychiatric Interview [25,26] was used to evaluate changes in major depressive disorder diagnosis and to characterize the sample in terms of comorbid anxiety disorders at baseline. The QIDS-C [28] was used to assess evaluator-rated depression symptom severity. The PHQ-9 [44] was used to evaluate self-reported depressive symptom severity. Finally, the Generalized Anxiety Disorder 7-item scale (GAD-7) [45,46] was used as a secondary outcome evaluating general anxiety symptom severity.

A semistructured interview designed by this research team was used to gather participant feedback regarding the intervention components. Feedback was also solicited via online self-reports at each assessment, and coaches recorded feedback received during the coaching sessions. Usage data were transmitted from the mobile phone to the lab’s secure server via encrypted, password-protected tunnels, and from the website using secure sockets layer (SSL). The number of times participants logged into the website was calculated by considering all site activity occurring within the same hour as corresponding to a single login.

### Analysis

Accuracy of the individual patient learner models was evaluated after the trial using Weka’s cross-validation [47] procedure. This procedure estimated the classification accuracy that would result from using models built from the complete labeled sensor datasets. In 10-fold cross-validation, the superset of all self-reported values for a particular state are paired with their concurrent sensor values and divided into 10 subsamples. One of the subsamples is the validation set, and the other 9 are combined to form the training set. A new model is generated using the training set. Next, the new model predicts states from sensor readings in the validation set, and these predictions are compared against the actual self-reported states to calculate the rate of accurate classifications (ie, the number of correctly predicted states divided by the total number of cases in the validation set). For regression trees, a correlation was calculated between predicted and actual values in the validation set.

This process is repeated 9 times, such that each of the other 9 subsamples is used once as the validation set. The estimator for the models is the average of these 10 accuracy rates. Next, bias corrected and accelerated 95% confidence intervals were constructed around the average accuracy rates for each state across participants, using 1000 bootstrap samples in SPSS 19 (IBM Corporation, Somers, NY, USA). For states predicted using regression trees, accuracy was calculated by averaging the correlations between predicted and actual values across participants.

Continuous outcome measures (ie, the PHQ-9, QIDS-C, and GAD-7) were modeled against time on an intent-to-treat basis, using mixed models for repeated measures. These models were chosen because they can handle a certain amount of nonrandom missing data [48]. Adding the slope of time as a random effect did not significantly improve log likelihood values over using only a random intercept; thus, only a random intercept was included to allow for individual variation between participants. Generalized estimating equations logistic regression was used to model the binary repeated outcome measure of presence versus absence of major depressive disorder diagnosis. These analyses were run using SAS 9.2 (SAS Institute, Cary, NC, USA) with restricted maximum likelihood methods.

## Results

### Participant Characteristics

**Figure 5:**
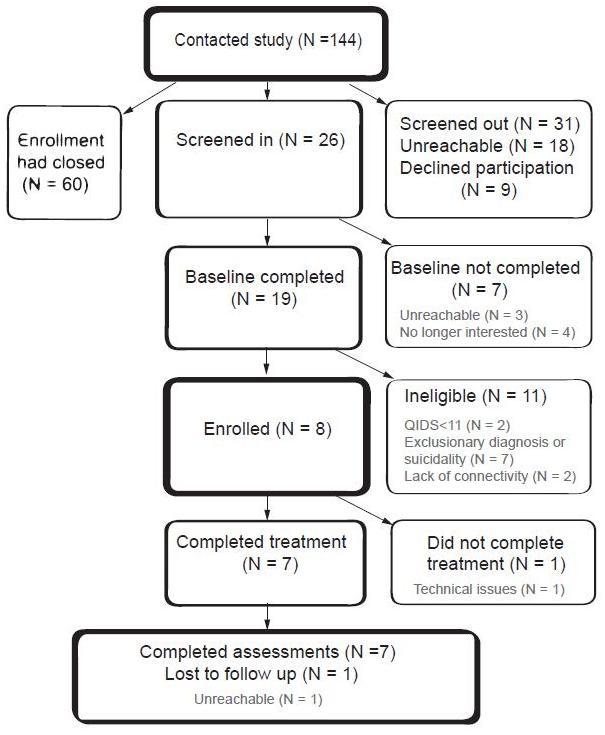
Flow of participants through the trial (QIDS = Quick Inventory of Depression Symptoms-Clinician Rated)

We enrolled 8 participants (7 females and 1 male) in the trial (see Figure 5). Ages of participants ranged from 19 to 51 years, mean 37.4 (SD 12.2). All participants reported at least a high school education, completing on average 14.3 years of education (SD 2.3). One participant (13%) identified as Hispanic and Caucasian, while the other 7 (88%) identified as non-Hispanic Caucasians; 7 (88%) were married and 1 (13%) was single.

Three of the participants (38%) were diagnosed with comorbid generalized anxiety disorder, but no other anxiety disorders, at baseline. Another participant (13%) was diagnosed with both generalized anxiety disorder and social phobia, and 2 other participants (25%) were diagnosed with generalized anxiety disorder, social phobia, and agoraphobia in the absence of panic disorder.

### Adherence

Seven participants completed all 8 weeks of participation, while 1 dropped out in week 3 due to technical problems using the phone and website.

#### Mobile Phone Training

On average, the 8 participants trained their phone 15.3 times (SD 8.3) during the first week of the intervention, dropping to 9.0 ratings (SD 6.5) during the fourth week, and 4.8 ratings (SD 4.6) during week 8. The participants reported that they would have trained the phone more often, had they received more prompts to label their states. Problems with network connectivity were encountered that reduced the number of prompts from the Web server that reached the phone, and at least 1 of the 7 treatment completers never received ecological momentary intervention. During the trial, we corrected this issue by storing ecological momentary intervention logic and the dates/times for phone prompts locally, on the phone itself. This way, participants did not require connectivity to receive prompts to train the phone or ecological momentary intervention.

#### Website Use

The 8 participants logged on to the website an average of 7.9 times and completed 4.8 out of 9 lessons over the trial. Website use may have been reduced due to technical problems with some of the tools and in rolling out new lessons to participants in the first half of the trial.

### Accuracy of Predictions

Across the 7 treatment completers, the mean rate of accurate classification for location was 60.3%, bootstrap 95% CI 43.2–77.2. The other categorical variables were difficult to compare across participants, as separate binary models were created for each available category. This was due to users’ ability to endorse more than 1 option (eg, “friends” and “family” can simultaneously occupy one’s immediate environment). As not all users reported ever being in certain categories, the existing models differed between users. The categorical states that were endorsed at least once by all treatment completers are listed in Table 1. 

**Table 1:** Mean accuracy indicators for machine learning models of categorical states common to all treatment completers (N = 7)

Model	Mean accuracy (%)	95% CI^a^
Location	60.3	43.2–77.2
Alone in the immediate vicinity (Y/N^b^)	80.1	76.2–84.5
Friends in the immediate vicinity (Y/N)	90.8	84.3–95.7
Alone in the larger environment (Y/N)	72.6	61.0–82.8
Miscellaneous people in the larger environment (Y/N)	90.9	83.8–97.3
Having a casual conversation (Y/N)	66.1	54.0–77.6
Not conversing (Y/N)	64.5	58.4–70.3

^a^ Bias corrected and accelerated 95% confidence intervals (CIs) using 1000 bootstrap samples.

^b^ yes/no.

The models for the scale-based states were not as encouraging. The cross-validation correlation coefficients were negative for all these states. Many participants’ regression trees predicted 1 particular value regardless of sensor data. Thus, at first glance one would expect the correlation coefficients to be near zero across all the participants. However, overall mood ratings were negatively skewed (skewness –0.49, SE 0.10), and this caused the average value gleaned from the training folds to produce outlying, negative deviations between the predicted and labeled values when high-labeled values were present in the validation dataset. The correlation coefficients are therefore not reported, as they are somewhat misleading; rather, the accuracy of the scale-based state models should be conceptualized as no better than chance.

### Clinical Outcomes

Per-protocol clinical outcomes are displayed in Table 2. The participant who dropped out failed to complete self-report measures at weeks 4 and 8, as well as interview measures at week 8. Another participant remained in treatment, but failed to complete interview assessments at week 4. However, all participants were included in the following analyses. Intent-to-treat analyses revealed that depressive symptoms self-reported on the PHQ-9 decreased significantly over time, *t*_13_ = 7.02, beta_week_ = –.82, *P* < .001. Evaluator-rated depressive symptoms on the QIDS-C also improved, *t*_13_ = 8.22, beta_week_ = –.81, *P* < .001. Over the course of treatment, participants also became less likely to meet diagnostic criteria for Major Depressive Disorder, *Z* = 2.15, b_week_ = –.65, *P* = .03. Scores on the GAD-7 indicated anxiety symptoms also decreased, *t*_13_ = 4.59, beta_week_ = –.71, *P* < .001.

**Table 2:** Per-protocol clinical outcomes

Variable	Baseline	Week 4	Week 8	Pre–post Cohen *d*^a^
% with MDD^b^	100% (8/8)	28.57% (2/7)	14.29% (1/7)	–
Mean (SD) PHQ-9^c^ score	17.13 (3.80)	9.00 (4.47)	3.57 (4.12)	3.43
Mean (SD) QIDS-C^d^ score	13.75 (2.71)	7.14 (3.89)	3.43 (3.10)	3.55
Mean (SD) GAD-7^e^ score	15.50 (4.72)	9.43 (4.04)	6.43 (2.30)	2.58

^a^ Standardized mean difference divided by the average of the baseline and week 8 SD.

^b^ Major depressive disorder diagnosis on the Mini-International Neuropsychiatric Interview [25,26].

^c^ Patient Health Questionnaire-9 [44].

^d^ Quick Inventory of Depression Symptoms–Clinician Rated [28].

^e^ Generalized Anxiety Disorder 7-item scale [45].

### Participant Feedback

Posttreatment, the 7 treatment completers rated their satisfaction with the mobile phone in general by agreeing or disagreeing with the statement “I am satisfied with it.” Their average rating was 5.71 (SD 1.38) on a scale of 1 (representing strong disagreement) through 7 (representing strong agreement). The most common problems for which participants sought technical support were loss of connectivity, shortness of battery life, and phone freezing during use. Connectivity difficulties were further evidenced on the feedback measures; several participants indicated they were never prompted to train the phone. Similarly, we received reports of failure to receive mood predictions and scheduled activity reminders. 

Despite these difficulties, during coaching calls 6 of the 7 treatment completers (86%) indicated the intervention was helpful in understanding triggers for negative moods and increasing their ability to recognize and modify distressing behaviors and cognitions. Two participants attributed these improvements to ecological momentary assessment on the mobile phone, while 4 of these participants cited ecological momentary assessment as well as the website. On the interview feedback measure, 1 of the participants stated that receiving the mood predictions was the most helpful component of the intervention. Another participant indicated that, because the predictions were helpful, she would have liked to continue using the mobile phone application after the study was over. Participants’ suggestions included lengthening the intervention and adding additional features such as a blog, messaging with coaches, or a recording tool to allow verbal elaboration on states when training the phone.

## Discussion

To our knowledge, we have created the first ecological momentary intervention and the first context-sensing system for depression. While we encountered some technological difficulties, patients who used the Mobilyze! intervention showed significant clinical improvement and reported a high level of satisfaction with the intervention. This system holds great promise in offering an entirely new depression treatment option with the ability to maintain continuous contact with the patient in his or her environment, recognize the patient’s behavior and states, and intervene accordingly. While we encountered numerous challenges, there were also many lessons learned, which we will discuss in detail below.

We will discuss the following problems, lessons, and future directions. We encountered problems with connectivity and battery drainage, which can be addressed through more effective management of the mobile application. Context sensing requires adequate user-labeled data, accurate and meaningful sensor data, and appropriate data mining algorithms; we identified potential improvements in each of these areas. Finally, our experience suggests several options for maximizing the clinical utility of model predictions. 

### Management of Mobile Phone Applications

We encountered numerous technical problems related to battery drainage and connectivity. We followed the XMPP specification requiring a continuous connection between interfacing devices. Thus, the mobile phone continually searched for a connection to the backend server. This exacerbated battery drain by the phone application, particularly when the network connection was weak. A potential fix is to use a different underlying network protocol. For example, we may have been able to implement the same functionality using binary SMS messages instead of XMPP. However, this would be a less extensible solution, as not all platforms support this feature. Rather, we will minimize this problem in the future by equipping the application with a battery management system. This functionality would reduce phone communication with the backend server when low battery power or weak network connectivity is detected. Developing onboard data processing applications that use compression and feature extraction to reduce the quantity of data shipped to backend servers may also improve power efficiency, as can delaying transmission of sensor data that are not immediately needed to times when the phone is charging.

In the future, we will explore additional methods of maximizing battery life. Continuous sensing imposes an “energy tax.” Applications that manage the frequency of data acquisition from energy-expensive sensors such as GPS can improve battery life. Adopting duty cycling techniques, which manage the sleep cycle of sensors to balance the sensing frequency and battery consumption, will promote technical efficiency [49]. In other words, system management must be smart enough to adapt to the local environment within the phone.

Disruption in connectivity sometimes prevented delivery of ecological momentary assessment prompts from the backend server to the user, thereby reducing the frequency with which users trained the phone. On discovery of this issue during the trial, dates and times for prompts to occur were generated and saved locally on the phone; however, participants may have already become accustomed to more infrequent labeling. For functions that do not require complex processing (which can drain the battery if performed on the phone), future applications should strive for as much connectivity independence as possible to ensure more reliable functionality and to reduce battery drain.

### Context Sensing

The ultimate aim of the machine learning system is to provide actionable evidence for outreach and other tailored patient interactions. To this end, our subsequent development process will focus on these semisequential stages: 1) increasing the amount of user-labeled data, across a greater variety of contexts, 2) improving accuracy and utility of sensor data, 3) improving accuracy of machine learning algorithms, and 4) maximizing the clinical utility of model predictions. The following section discussed each of these stages in more detail.

#### Increasing the Quantity and Variability of User-Labeled Data

One of our “known unknowns” is how well machine learning can extract the desired models from the types of sensor data we collected. This is an unknown because there was an insufficient amount of user-labeled data, and this contributed to the negative correlation coefficients on the scale-based states. Reinforcement techniques, such as those used successfully in the video game industry, could increase the frequency with which users label their states and reduce influence of outliers. Chris Bateman, a well-known game designer, described different types of rewards that motivate continued use of games [50]. Many of these would easily translate to Mobilyze!. For example, “currency rewards” could be used to reinforce training the phone by providing patients with points that can be used for shopping (eg, for new music files to play on their phone). “New toys” could also be used to promote adherence to ecological momentary assessment, by offering users new capabilities (eg, more sophisticated graphs of their states, and the ability to create a new, individualized state to label and visualize) in exchange for training the phone. “Rank rewards” can be as simple as informing users they have increased in rank (eg, “novice,” “expert,” or “master”) after they train the phone a set number of times.

Due to the preliminary nature of this study, model accuracy was evaluated retrospectively and through manual requests to the Weka software. This limited our ability to conduct ecological momentary assessment in an adaptive manner that would improve each user’s particular models. Our software will be adapted to interface with Weka, such that accuracy is automatically monitored throughout the trial. Accuracy measurements will be used to guide the ecological momentary assessment, as users can be queried more often on states for which their models are less accurate. Adherence to ecological momentary assessment procedures may be reinforced by communicating accuracy measurements or improvements to the user, such that users can track their progress in training the phone. This kind of reinforcement can be conceptualized as “completeness,” as some users are motivated to strive for perfect scores [50].

In addition to an inadequate quantity of labeled data, these data did not exhibit variability across the entire range of measured states. This may have occurred for several reasons. Users probably did not train the models across their entire set of contexts due to differential levels of convenience or social norms regarding use of mobile phones. Another possibility for low variability is that there actually was little variability in certain states, at least as detectable via the Mobilyze! ecological momentary assessment.

In an informed approach, variability of the user-labeled states used to construct each model can be harnessed to evaluate whether the user experience can be improved. The rate, sensitivity, and quality of user prompts can be modified accordingly. For example, users could be prompted to complete a “scavenger hunt” and train the phone when previously invariant states change (eg, a user who has always reported a sedentary state could be specifically prompted to train the model the next time they are more physically active). State labeling queries could also be recalibrated for greater sensitivity. Such adaptations can be automated to occur in response to individual differences in variability. For example, if an individual almost always reports being at home, the location query could be changed to allow specification of the particular room of the home. In a similar vein, it is likely that there are more variable states unique to a given user (eg, a user may report relatively static overall mood, but experience somatic symptoms of depression that vary quite a bit). In the future, the Mobilyze! system will allow users to create individually tailored states. Indeed, some participants in this trial indicated the Mobilyze! ecological momentary assessment did not query all the states they believed to be important. Finally, automated checks on label skewness can be used to encourage users to label their states more often when they are experiencing underreported values.

#### Improving Accuracy and Utility of Sensor Data

Accurate context sensing is fundamentally dependent on the quality of the sensor data and the quality of the features derived from the sensor data. Our experience has taught us that sensor data are often inaccurate, or technically accurate but misleading. For example, the ambient light sensor reported values brighter than the maximum meaningful value, and the accelerometer produced seemingly contradictory data when the phone was dropped or swinging as it was carried in a bag. To be functionally predictive, the raw sensor data should first be accurate from a technological perspective and then, if possible, be manipulated to extract features that describe more actionable, meaningful data [35]. An example of removing technological errors would be to discard implausible GPS coordinates. For the accelerometer, a feature could be extracted by using a time series of readings to determine trajectory of the phone and classify it as likely to be “swinging,” “dropping,” or neither. Our more detailed plans for improving the quality of sensed data can best be illustrated by specific examples, which we will describe here in terms of the GPS data. GPS was anticipated to predict location, and yet the data proved so unreliable that the learning algorithm included GPS coordinates in only 1 of the treatment completers’ location models.

Mobilyze! generates 2 sets of GPS data. One set comprises GPS readings sampled every 5 minutes, provided users had adequate telecommunications connectivity to enable GPS detection. This set does not include instances during which users trained the models. The second, much smaller set of GPS data consists of GPS readings at all physical locations where users labeled their location.

Accurate data in both datasets are critical for different reasons. Labeled GPS data are used to generate the models, which are designed to compare accurate data with accurate labeled states. If these data are compromised, the models themselves become unreliable. The second, unlabeled GPS dataset will ultimately be used to launch intervention. If these data are inaccurate, intervention may be triggered at inappropriate times. Given its completeness, the unlabeled dataset can also provide a higher-resolution perspective on the accuracy of the GPS transponder and users’ daily GPS trajectories.

The following sections describe techniques we used to clean and understand GPS data post hoc. With appropriate engineering, however, these techniques could be used in real-time scenarios. As no single technique of cleaning and reformatting data is likely to effectively resolve all issues, a strategy combining multiple approaches should be used to improve accuracy. For the purposes of this description, we will use the location data provided by a project staff member due to her ability to confirm her location and travel patterns. Her GPS data were exported into Keyhole Markup Language (a standard method of defining geographic path information) and visualized on Google Earth.

##### Technological Error Exclusion

On several occasions data reflected visibly impossible scenarios, depicting GPS values that were up to 15 miles away from actual user-reported positions. Values that are impossible based on user-reported geographic position can be excluded. For example, we removed any values that depicted the staff member in a body of water, once she confirmed she had never been on the water. In the future, real-world geographic information system and commercial databases could be used to automate this process. For example, GPS readings could be compared against topographical features, and users could be queried to confirm less probable scenarios (eg, GPS indicates the user is in a remote area with extremely low population density, on a mountain, or in a body of water).

Other future techniques could exclude GPS points that suggest a user has traversed a greater than probable distance within a particular amount of time (eg, >70 miles/hour). Here, unlabeled data may be used to clean labeled data. Unlabeled GPS coordinates obtained before or after labeled coordinates can be used to calculate the implied velocities. Long term, this would be a way to leverage chronological GPS data to assist the learners. This technique could also prevent intervention based on improbable GPS data, regardless of whether users have recently labeled their states.

##### Feature Extraction

Some data inaccuracy is likely caused by the limited resolution of the GPS transponder, which may result in GPS coordinates that are inaccurate but relatively close to the user’s actual location. Based on observations of data reported by the phone platform, GPS data were accurate in some cases to the 8- to 15-m^2^ approximate resolution (if edge cases are removed). This range may not always be effectively actionable, and yet uniformly excluding unlikely values may not be the best solution. Feature extraction may help to salvage some of these GPS readings.

For example, users could be asked to predefine geographical places of significance or high frequency (“hot spots”) at the beginning of the intervention using freely available geographic information system databases. A set of coordinates would thus be predefined and, in addition to raw GPS data, a “distance-from-hot-spot” feature could be added to enhance the quality of the GPS data. For example, a user may be at home, but the GPS data are fluctuating within a 50-m radius from the “home” hot spot. The feature would consist of the calculated distance from the home hot spot. Further, if this distance is within the phone’s estimated resolution range, a binary feature could be defined to indicate that the user is likely to be at home.

Sensors could also define a user’s physical trajectory. By storing a set of recently logged GPS positions in an external dataset, a spatial model could describe a best-fit line that estimates users’ trajectory. This best-fit line could be used to generate a feature for the “estimated GPS” coordinates. This may be particularly helpful to handle missing data points occurring when the phone cannot obtain GPS readings due to connectivity problems.

Many other features could be envisioned. New features could be constructed based on interactions between sensors or between predictions generated by other models. For an example of the latter, being at home and alone may be depressogenic, while being at home and with friends may predict positive mood. Thus, the interaction between predictions for location and relationship to others in the environment could be inserted into the model predicting mood. There are also algorithms that use combinations of mathematical functions to automatically extract features from raw sensor datasets, and select or prune features based on their impact on learner accuracy [51,52].

#### Improving Accuracy of Learners

Although accuracy of the categorical states was promising, the accuracy of scale-based state models was poor and merits further discussion. On further examination of the scale-based state models, there appear to be at least 3 ways in which their accuracy might be directly improved. First, transformations can be used to normalize the distribution of user-labeled ratings prior to model generation and accuracy testing. Second, regression tree pruning can be selectively disabled. Regarding this option, we found post hoc that when regression trees were generated without pruning, models may be more likely to include sensor values rather than predicting a constant value and ignoring sensor data. Third, the system should be improved more generally to use ensemble methods. For example, bootstrapping or selective weighting can be applied to the labeled sensor data, generating multiple new training datasets [53,54]. Next, a model is created from each of these datasets using 1 of the learning algorithms (eg, decision trees). For each incoming set of sensor data, a prediction would be calculated by each of these models. The final prediction would be an aggregate of these individual predictions. Aggregation is likely to result in greater accuracy than use of individual models [55].

#### Improving Clinical Utility of Predictions

As we were unable to anticipate the accuracy with which users’ states would be predicted in this field trial, predictions were not used to trigger intervention; rather, they were simply displayed to the user. Once models meet particular accuracy benchmarks, future studies will integrate them more fully into clinical applications. To leverage the potential benefits of a mobile platform, we will iteratively examine the methods by which feedback and outreach are delivered. For example, if outreach is delivered suggesting use of a particular tool, pre- and post- mood ratings could be used to evaluate its success for the user in that context. Experimental designs could also be employed to evaluate the impact of more sophisticated forms of feedback. We have further developed the Mobilyze! website such that it can display the GPS sensor readings on Google Earth. We aim to create a “mood map,” where all GPS coordinates at which users labeled their mood will be displayed as colored dots. These dots will be color coded to convey the mood rating at each point, such that users can better visualize the impact of physical location on their mood.

### Process Lessons

When we began designing this project in late 2008, Nokia’s Symbian operating system and the Android operating system were our only options in terms of multitasking mobile phone platforms. It appeared more prudent to choose Symbian due to its longer history and platform maturity, whereas Android had just been deployed in October 2008. However, by 2010 Android became the most frequently purchased system in the United States [56], and Nokia has announced plans to retire the Symbian operating system in favor of Windows Phone 7 [57]. This speaks to the rapidity of development and change of technology used in mobile applications. In the future, we will use a cross-platform development framework (eg, Nitobi’s PhoneGap) capable of deploying applications to several major mobile phone platforms, which should allow greater adaptability to this changing market. This would also allow individuals who already possess compatible smartphones to download the Mobilyze! phone application to their own phones, thus mitigating the costs of providing smartphones to users, as well as the inconvenience for users who would otherwise need to either carry 2 phones or transfer their existing phone settings to a new phone.

While we have learned much from this initial development enterprise, we have also found the numbers of challenges impossible to manage within the scope of a single project. These challenges included the cross-disciplinary expertise needed to process the collected data and the time invested to ensure basic functionality of ecological momentary intervention and website components. Investment of effort into the other intervention components resulted in limited resources to investigate the myriad techniques that could be used to clean and reformat the sensor data or improve the machine learning methods. Current plans are to continue research and development in a staged sequence, beginning with improvement of state predictions in the absence of any clinical intervention or website. Clinical intervention components will be added after the machine learning components function adequately.

### Other Limitations

The current trial did not include a control condition. Once Mobilyze! is refined as previously described, a randomized controlled trial will be used to determine whether clinical improvements can be attributed to Mobilyze! as opposed to regression to the mean or other confounding factors. Further, Mobilyze! is a multilevel intervention comprising more traditional Internet approaches to behavioral intervention (ie, didactic lessons, exercises), coaching, ecological momentary assessment, and ecological momentary intervention. Factorial designs will be required to isolate the specific contributions of each of the components of Mobilyze! [58,59].

### Conclusion

This trial demonstrated the feasibility, appeal, and potential efficacy of a new paradigm for mobile intervention targeting depression. The website delivered behavioral skills training, while the mobile phone provided self-monitoring with tailored, real-time feedback and intervention. Our initial experience suggested the feasibility and utility of a context-aware system using mobile phone sensing capabilities. We have described the numerous lessons learned and areas for continued development.

Mark Weiser, the father of ubiquitous computing, wrote that “The most profound technologies are those that disappear. They weave themselves into the fabric of everyday life until they are indistinguishable from it” [60, p. 94]. A context-aware system that can sense a person’s behavior and state, coupled with a treatment platform that can positively reinforce adaptive behaviors and provide support for changing those that contribute to depression, has the potential to provide a fundamentally new model for mental health intervention.

## Multimedia Appendix 1

List of all collected phone sensor data

## Abbreviations

GAD-7: Generalized Anxiety Disorder 7-item scale
GPS: global positioning system
PHQ: Patient Health Questionnaire
QIDS-C: Quick Inventory of Depression Symptoms–Clinician Rated
SMS: short message service
XMPP: extensible messaging and presence protocol
