# Supplementary material for: Harnessing Context Sensing to Develop a Mobile Intervention for Depression
Source: J Med Internet Res. 2011 Aug 12;13(3):e55. doi: 10.2196/jmir.1838 (PMC3222181; doi:10.2196/jmir.1838)
Supplement: Supplementary file 1 [file jmir_v13i3e55_app1.pdf]

Multimedia Appendix 1

Acceleration: X Axis
Acceleration: Y Axis
Acceleration: Z Axis
Altitude
Ambient Light
Application Status (multiple)
Battery Level
Bluetooth (multiple)
Cellular Tower
Country
Current Application (multiple)
Current Area Code
Current Mobile Country
Current Mobile Network
Current Profile
Day and Night Sensor
Day of Month
Day of Week
Device Orientation
Device Proximity
Language
Last Call Duration
Last Caller
Last Initiated Call Duration
Last Received Call Duration
Latitude
Lock Status
Longitude
Missed Call Count
Power State
Ring Volume
Rotation: X Axis
Rotation: Y Axis
Rotation: Z Axis
Time of Day
Time Since Last Call
Unread E-Mail Count
Unread SMS Count
